# Patient safety with covid-19 in hospital units: a scoping review

**DOI:** 10.1590/0034-7167-2022-0557

**Published:** 2023-10-09

**Authors:** Fernanda de Macedo Coelho Leite, Eloysa dos Santos Oliveira, Bruna Vilar Soares da Silva, Evelin Beatriz Bezerra de Melo, Rodrigo Assis Neves Dantas, Daniele Vieira Dantas

**Affiliations:** I Universidade Federal do Rio Grande do Norte. Natal, Rio Grande do Norte, Brazil

**Keywords:** Health Services, Patient Safety, Hospital Units, Coronavirus Infections, Covid-19, Servicios de Salud, Seguridad del Paciente, Unidades Hospitalarias, Infecciones por Coronavirus, Covid-19, Serviços de Saúde, Segurança do Paciente, Unidades Hospitalares, Infecções por Coronavírus, Covid-19.

## Abstract

**Objective:**

Map, in the scientific literature, the actions taken to promote the safety of patients with covid-19 in the hospital context.

**Methods:**

This is a scoping review according to the Joanna Briggs Institute, using the Checklist Preferred Reporting Items for Systematic Reviews and Meta-Analyses Extension for Scoping Reviews. In April 2022, searches were performed on nine data sources. The results were summarized in a table and analyzed descriptively.

**Results:**

Fifteen studies were selected to compose the final sample. Most articles refer to cohort studies, followed by clinical trials. As for the areas of activity, there was a predominance of surgical centers, followed by adult and pediatric Intensive Care Units.

**Conclusions:**

With this review, it was possible to map measures such as contingency plans and reorganization of beds, rooms, and operating rooms, in addition to the isolation and distancing practiced by patients and professionals.

## RESUMO

**Objetivo:**

Mapear, na literatura científica, as ações adotadas para promover a segurança do paciente com covid-19 no contexto hospitalar.

**Métodos:**

Trata-se de uma revisão de escopo de acordo com o Instituto Joanna Briggs, utilizando o *Checklist Preferred Reporting Items for Systematic Reviews and Meta-Analyses Extension for Scoping Reviews*. Em abril de 2022, foram realizadas buscas em nove fontes de dados. Os resultados foram sintetizados em um quadro e analisados de forma descritiva.

**Resultados:**

Foram selecionados 15 estudos para compor a amostra final. A maioria dos artigos se refere a estudos de coorte, seguidos de ensaios clínicos. Quanto às áreas de atuação, notou-se predominância dos centros cirúrgicos, seguidos por Unidades de Terapia Intensiva adulto e pediátrica.

**Conclusões:**

Com esta revisão, foi possível mapear medidas como planos de contingência e reorganização de leitos, quartos e salas de cirurgias, além do isolamento e distanciamento praticados pelos pacientes e profissionais.

## RESUMEN

**Objetivo:**

Mapear, en la literatura científica, las acciones adoptadas para promover la seguridad del paciente con covid-19 en el contexto hospitalario.

**Métodos:**

Se trata de una revisión de ámbito de acuerdo con el Instituto Joanna Briggs, utilizando el *Checklist Preferred Reporting Items for Systematic Reviews and Meta-Analyses Extension for Scoping Reviews*. En abril de 2022, fueron realizadas búsquedas en nueve fuentes de datos. Los resultados fueron sintetizados en un cuadro y analizados de manera descriptiva.

**Resultados:**

Fueron seleccionados 15 estudios para componer la muestra final. La mayoría de los artículos se refieren a estudios de cohorte, seguidos de ensayos clínicos. Cuanto las áreas de actuación, notado predominancia de los centros quirúrgicos, seguidos por Unidades de Cuidados Intensivos adulto y pediátrica.

**Conclusiones:**

Con esta revisión, fue posible mapear medidas como planes de contingencia y reorganización de lechos, cuartos y salas quirúrgicas, además del aislamiento y distanciamiento practicados por pacientes y profesionales.

## INTRODUCTION

In December 2019, in the city of Wuhan, China, severe cases of pneumonia associated with a new coronavirus, identified as Severe Acute Respiratory Syndrome Coronavirus 2 (SARS CoV 2), were detected. Due to the high transmissibility of the virus and the exponential number of infected people worldwide, in 2020 the World Health Organization (WHO) declared a pandemic resulting from the disease called CORONA VIRUS DISEASE-19 (covid-19), caused by the new coronavirus. Since then, the first measures aimed at controlling the course of the disease began to be publicized, such as the closure of borders^(1-3)^.

Despite being a worldwide phenomenon, it is worth noting that the covid-19 pandemic can develop very differently depending on the peculiarity of each place, such as the availability of infrastructure, professionals, environments and practices; that is: the impacts of the pandemic are not homogeneous, so a more critical view of this process is necessary^(1,4)^.

In this sense, one can evaluate the Brazilian State, which, unlike developed countries, has hospital networks with fragile structures, lacking reinforcements and constant adaptations. This could be seen in the pandemic, in which infirmary beds had to be transformed into beds in the Intensive Care Unit (ICU) for the care of critical patients with covid-19. Such emergency changes may also compromise patient safety^(1,4)^.

It should be noted that the escalation of the disease was unprecedented and the increase in cases was accompanied by the unimaginable growth in the number of deaths, as well as the overcrowding of beds, scenes of saturation of health services, isolation of patients, death without goodbyes. and collective burials^(1,5)^. Since then, until the first half of 2022, covid-19 has had more than 500 million cases and 6 million deaths worldwide^(6)^.

At the same time, it is known that Patient Safety, according to the National Patient Safety Program (PNSP), proposes measures to prevent and reduce incidents in health services, promoting, for example, training processes in patient safety for managers or professionals acting directly or indirectly in health care that results in unnecessary harm to the user^(7-8)^.

In this context, due to the high transmissibility of covid-19 by contact and droplets, the pandemic brought new challenges to health institutions. As a result, the performance of the PNSP was essential in view of this reality, since it required, for example, a quick redefinition of service flows and the creation of new protocols, to offer safe assistance^(9)^. However, several countries, including Brazil, experienced the reality of a shortage of qualified professionals to provide care to users in critical condition due to covid-19, directly compromising patient safety^(4)^.

Added to this, Prado et al. (2021)^(4)^ emphasize the existence of other factors that need to be considered in order to provide safe care. Examples of these factors are the production and proper distribution of ventilators and personal protective equipment (PPE) and access to essential equipment for care, highlighting the need to use them safely. The scarcity of these resources, therefore, was a present reality that threatened the safety of professionals and patients.

In addition, the safety culture and the set of skills developed within the scope of patient safety, according to the PNSP, are essential for adapting to a constantly changing environment. In this way, covid-19 requires the work of responsible teams as essential tools for the success of a program of change and support for patients, professionals and organizations^(10)^.

In view of the above, it is clear that the pandemic scenario has led to an urgent need to reorganize care practices, especially with regard to patient safety and covid-19. Therefore, this study is justified by its relevance in bringing contributions to the scientific, academic and health professionals’ community regarding the presentation of data from the scientific literature on patient safety in the context of the covid-19 pandemic in the hospital environment. The purpose was to promote a technical-scientific basis on the subject, in addition to identifying gaps, enabling the development of new studies on the subject.

## OBJECTIVE

Map, in the scientific literature, the actions taken to promote the safety of patients with covid-19 in the hospital context.

## METHODS

This is a scope review, which aims to identify gaps in knowledge when exploring concepts in a given area, providing the synthesis and dissemination of the results obtained. The development of the study and the elaboration of the protocol and flowchart followed the recommendations of the JBI^(11)^, using the Checklist Preferred Reporting Items for Systematic Reviews and Meta-Analyses Extension for Scoping Reviews (PRISMA-ScR)^(12)^. Furthermore, the article was registered on the Open Science Framework (OSF) platform. (osf.io/b3m9t/).

Furthermore, for the elaboration of the study, five steps were followed: 1) construction of the research question; 2) identification of records relevant to the study; 3) selection and inclusion of studies; 4) data summarization; 5) summary of results^(13)^.

For the formulation of the research question, the mnemonic Population, Concept and Context (PCC) was used, being P - patients with covid-19; C - promotion of safe care; C - hospital units^(12)^. In this way, the following research question was defined: “What actions are taken in hospital units to promote the safe care of patients with covid-19?”

In order to identify the similarity of studies with the present review, a search was carried out in the Open Science Fromework (OSF) data sources; JBI Clinical Online Network of Evidence for Care and Therapeutics (COnNECT+), Database of Abstracts of Reviews of Effects (DARE), The Cochrane Library, as well as the International Prospective Register of Ongoing Systematic Reviews (PROSPERO), in which no similar results were found.

The descriptors were selected according to the Descriptors in Health Sciences (DeCS) e *Medical Subject Headings* (MeSH), to follow the standard terminology of concepts in Portuguese and English Coronavirus/*Coronavirus Infections*; Segurança do Paciente/*Patient Safety*; Gestão da Segurança/*Safety Management*; Unidades Hospitalares/*Hospital Units*; Serviços de Saúde/*Health Services*. Keywords were not used. It is also important to emphasize that limitations found regarding the controlled descriptors and keywords are justified by a certain scarcity of these terms in scientific articles, since they started to be published after the beginning of the pandemic.

In the elaboration of the search syntaxes, the Boolean operators “AND” and “OR” were used, being adapted according to the particularity of each data source, conserving the combinations between the descriptors. The crossings performed, in order to align the Boolean operators with the PCC, are described in Chart 1.

**Chart 1:** Descriptors and keywords used in the search, 2022

PCC	MeSH		*Keywords*
Population	Coronavirus Infection	OR	
AND			
Concept	Patient Safety OR Safety Management	OR	
AND			
Context	Hospital Units OR Health Services	OR	

In April 2022, searches were performed in nine data sources: Cumulative Index to Nursing and Allied Health Literature (CINAHL), Cochrane library, Latin American and Caribbean Literature in Health Sciences (LILACS), National Library of Medicine and National Institutes of Health (PUBMED), Scientific Electronic Library Online (SciELO), Science Direct, Elsevier’s Scopus, Web of Science and Wiley Online Library. The search syntaxes used in each data source are listed in Chart 2.

**Chart 2:** List of search syntaxes in data sources, 2022

Data sources	Search syntaxes
^*^CINAHL	(Coronavirus Infections) AND (Patient Safety OR Safety Management) AND (Hospital Units OR Health Services)
MEDLINE/PubMed	(“coronavirus infections”[MeSH Terms] OR (“coronavirus”[All Fields] AND “infections”[All Fields]) OR “covid-19”[All Fields]) AND (“patient safety”[MeSH Terms] OR (“patient”[All Fields] AND “safety”[All Fields]) OR “patient safety”[All Fields]) AND “hospital units”[MeSH Terms] OR (“hospital”[All Fields] AND (“units”[All Fields]) OR “hospital units”[All Fields])
^†^LILACS	Coronavirus Infections AND Patient Safety OR Safety Management [Palavras] and Hospital Units OR Health Services [Palavras]
Academic Google	(“Coronavirus Infections” OR “covid-19 “) AND (“Patient Safety” AND “Hospital Units”)
^‡^SciELO	(^*^Coronavirus Infections) AND (Patient Safety OR Safety Management) AND (Hospital Units OR Health Services)
Science Direct	(SU (Coronavirus Infections)) AND (SU (Patient Safety OR Safety Management)) AND (SU (Hospital Units OR Health Services))
^§^Scopus	TITLE-ABS-KEY (coronavirus AND infections) AND TITLE-ABS-KEY (patient AND safety OR Safety AND Management) AND TITLE-ABS-KEY (hospital AND units health AND services)
Web of Science	((TS=(Coronavirus Infections)) AND TS=(Patient Safety OR Safety Management)) AND TS=(Hospital Units OR Health Services)
Gale Academic OneFile	(Coronavirus Infections OR covid-19) AND (Patient Safety) AND (Hospital Units)
Wiley Online Library	“Coronavirus Infections” anywhere and “Patient Safety OR Safety Management” anywhere and “Hospital Units OR Health Services” anywhere

* CINAHL: Cumulative Index of Nursing and Allied Health; † LILACS: Literatura Latino-Americana e do Caribe em Ciências da Saúde; ‡ SciELO: Scientific Electronic Library Online; § Scopus: Elsevier’s Scopus.

The search process took place through the Journal Portal of the Coordination for the Improvement of Higher Education Personnel (CAPES) and through the Federated Academic Community (CAFe), a tool provided by the Federal University of Rio Grande do Norte (UFRN).

Ministerial ordinances, theses, dissertations, guidelines, and scientific articles were included, without time frame or language restriction. However, abstracts, opinion articles, letters to the editor and records that did not meet the proposed theme or duplicates were excluded - these having been considered only once.

The search and selection of studies was carried out simultaneously and on different devices by two independent and duly trained evaluators. In cases of divergence between the selected articles, a third evaluator was consulted to, after discussions, decide between the inclusion or exclusion of the study in the sample. To summarize the results, a table was prepared according to the study identification variables (type of study, level of evidence, country, year of publication, area of activity, safety measures/protocols adopted in the studies and outcome), analyzed descriptively.

Regarding the level of evidence and degree of recommendation, the established by the Oxford Center for Evidence-based Medicine^(14)^ was considered, when determining that the lowest number corresponds to a better level of evidence and the classification in “A” means higher recommendation, representing greater relevance for the scientific community.

## RESULTS

**Figure 1:**
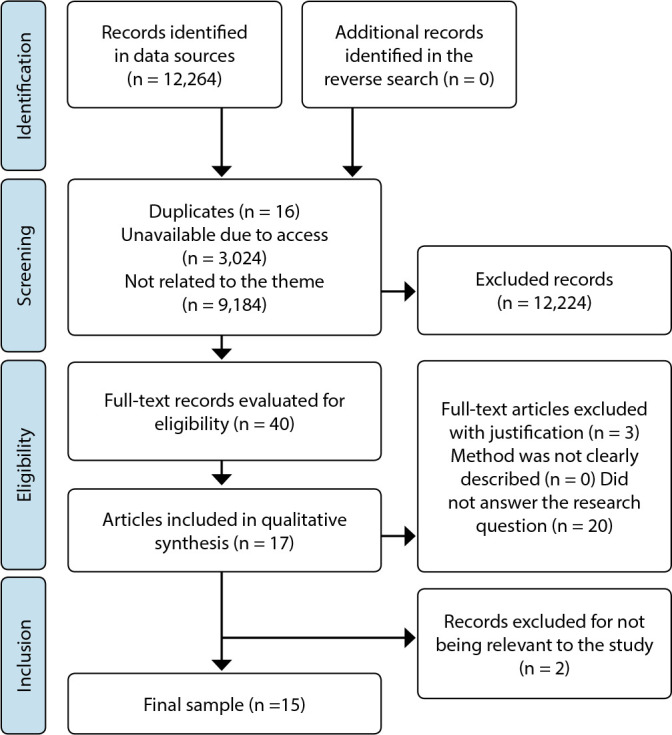
Search flowchart adapted from Preferred Reporting Items for Systematic Reviews and Meta-Analyses Extension for Scoping Reviews (PRISMA ScR), 2022

Through searches in data sources, 12,264 scientific articles were found, with 15 studies selected to compose the final sample, as shown by the steps in Figure 1.

As for the types of studies, seven (46.6%) of the records refer to cohort studies, with three (20%) clinical trials, two (13.3%) systematic reviews, one (6.6%) narrative review, a case-control study, and an institutional protocol.

Regarding the level of evidence of the studies, seven (46.6%) articles have level 2B; four (26.6%), level 1A; two (13.3%) have no evidence; one (6.6%) is level 1B; and one (6.6%) at level 3B. Furthermore, the countries with the highest number of publications were the United States of America (USA), with three (20%) studies, and Saudi Arabia, with two (13.3%). However, at a continental level, the present review identified a highlight in Europe, with six (40%) publications.

Concerning the areas of action in the health services where the studies were carried out, there was a predominance of surgical centers (26%), followed by adult and pediatric ICUs (20%). The other studies had a ward, a mental health hospital, a hemodialysis room, a sterilization material center (CME), an emergency department as a scenario, and another study was developed during a gastrointestinal endoscopy procedure.

Chart 3 displays the summary of studies referring to the final sample, according to the identification of the studies, type of study, level of evidence, country, year of publication, area of expertise, safety measures/protocols adopted in the studies and outcome.

**Chart 3:** Summary of the studies regarding their identification, type of study, level of evidence, country, year of publication, area of expertise, safety measures/protocols adopted in the studies and outcome, 2022

^*^ID	Study type/ ^†^LE/ Country/Year	Occupation area	Actions taken	Outcomes
Alsofyani et al.^(15)^	Institutional protocol / N / E / Saudi Arabia / 2020	Operating rooms and ICU	Use of PPE, bed contingency, active search, and monitoring of suspected cases among professionals and patients, admission screening with temperature measurement, health education and continuing education.	The study did not address the results regarding the implementation of the guidelines in the analyzed hospitals.
Cho et al.^(16)^	Cohort study/2B/ South Korea / 2020	hemodialysis	HD-positive patients: transferred to a hospital with an isolation room and a portable dialysis machine. Positive healthcare workers: admitted to a hospital with an isolation room, covid-19 care centers or home isolation.	During isolation, nine patients with symptoms tested negative for SARS-CoV-2. Two health professionals were diagnosed in the closure test.
Kawabata et al.^(17)^	Cohort study/2B Japan/2020	Gastrointestinal endoscopy	Use of a cube-shaped box on the patient’s head with two windows to allow endoscopy.	Patients suspected of having covid-19 were treated, and there were no complications. Professionals rated the device’s sense of security as 5/5.
Morgan et al.^(18)^	Clinical trial/1A/ USA/2020	Critical care unit of a children’s hospital	Among institutions, 91% have implemented changes to their inpatient COVID-19 emergency response systems, 76% have adhered to new protocols for patients with suspected COVID-19, and 74% are using new or adapted technologies.	Most pediatric institutions have adapted their care protocols. The changes were related to PPE, airway management, team members and professional and patient safety.
Rodriguez and Tantillo^(19)^	Systematic review/1A/ USA/2020	Mental health	Inpatient care was described using Gunderson’s five functions: containment, support and support, structure, involvement, and validation.	To prevent the spread of covid 19, one must be prepared to manage the potential increase in hospitalizations, with safety for patients and professionals, as well as adequacy of facilities.
Aljohani et al.^(20)^	Cohort study/2B/ Saudi Arabia / 2021	Bariatric surgery	Use of preoperative respiratory scoring system screening to select low-risk patients for elective surgery.	None of the patients developed postoperative complications or were admitted to the ICU. Only two patients were tested for covid-19, and both were negative.
Erbas e Dost^(21)^	Cohort study/2B/ Turkey / 2021	ICU	Use of aerosol box during percutaneous tracheostomy.	No healthcare professional was infected with SARS-CoV-2 while performing the tracheostomy procedure.
Huddy et al.^(22)^	Cohort study/2B/ UK/2021	Colorectal surgery	Creation of a “covid safe” robotic unit for major elective surgeries, minimized employee contact with possible covid patients and isolation of patients, in addition to performing swabs before surgery.	There were no known cases of patients included in the study developing coronavirus throughout the perioperative period.
Jachetti et al.^(23)^	Cohort study/2B Italy/2021	Emergency department	Separate screening, isolation for symptomatics, dedicated area for critical and non-critical covid patients, full PPE throughout, pre-isolation area for non-covid patients, decreased swab wait time.	Unfortunately, the changes and contingency plans were not enough and the emergency department remained overcrowded.
Landoas et al.^(24)^	Cohort study/2B/France/ 2021	Wards	Use of mask by health professionals and patients, hand hygiene, physical distancing, implementation of screen in double rooms and disinfection of shared equipment.	The safety measures adopted drastically reduced the nosocomial transmission of the virus.
Mihalj et al.^(25)^	Narrative review/‡n/e Switzerland /2021	Cardiac surgery	Postponement of non-emergency surgeries, telemedicine for preoperative evaluation, use of masks and PPE, patient swabs during triage, self-isolation before surgery, specific operating room, and nasal decolonization for patients with covid-19.	Operating on COVID‑19 patients may be reasonable in those who remain symptom-free. Nasal decolonization measures were causing more damage by increasing your risk of coughing or sneezing.
Penwill et al.^(26)^	Clinical trial/1A/ USA /2021	Children’s Hospital	Regular, transparent, multimodal, and two-way communication was optimized by team members. Physicians increased use of videoconferencing and telehealth.	Changes were identified such as: adoption of new hospital policies, video communication, personnel models, education strategies and support for the mental health of the team.
Pilosof et al.^(27)^	Case-control study/3B/ Israel/ 2021	Intensive care unit	The control of nosocomial infections was investigated under the supervision of the team. Reduction of medical errors through the adoption of protocols. Prevention of contamination by covid-19. Stimulation of teamwork and operations management.	Increased virtual visibility of patients and the team, optimization of the unit, facilitation of communication between the team and improvement of care through technological resources.
Martins, Xavier and Cobrado ^(28)^	Systematic review/ 2A/ Portugal/2022	Sterilization Material Center (CME)	Use of ethanol and isopropanol on porcelain and ceramic surfaces, alcohol, chlorhexidine, PVP-I and soap on biological surfaces, silver, copper, and aluminum oxide combined in air conditioning systems, in addition to UV-C light, heat with different humidity levels for cleaning PPE, ozone.	Alcohols and hand washing are the best options when it comes to choosing a quick and effective action against the virus. After decontamination, treatment with ozone is indicated.
Nahidi et al.^(29)^	Clinical trial/1A/ Australia/2022	Critical care unit of a children’s hospital	Of the nurses, 82.3% felt prepared to manage patients with covid-19; 93.4% received specific education, training, or instruction; 55.7% reported that the pandemic has increased their workload.	Most nurses reported being prepared to manage patients with covid-19. Those who had undergone training stood out in the answers.

* ID = Identification; † LE = Level of evidence; ‡ n/e = No evidence.

About the safety measures and protocols adopted in the hospital context, it is demonstrated that the most recurrent practices were those of contingency and reorganization of beds, rooms and operating rooms, as well as the isolation and distancing practiced by patients and professionals^(15-16,22-25)^.

It is worth mentioning that important security measures were rarely present, such as the active search and monitoring of suspected cases in professionals/patients and continuing education^(15)^, adaptation of the support staff to patients with covid-19^(23)^, changes in protocols, plans, technologies and policies in care^(18)^, in addition to care based on Gunderson’s five functions (restraint, support and support, structure, involvement and validation)^(19)^.

The repercussions of the adopted measures were almost totally positive, being evident the situations in which the patients did not have commitments or complications in the procedures; there was also promotion of greater safety in surgical procedures^(22,25)^ and implementation of effective communication based on new hospital practices to provide more qualified assistance^(17,20,26-27)^. Other examples of optimistic outcomes were shown in studies whose results showed a reduction in virus transmission^(24)^, including studies that showed professionals and patients with no record of infection during the study^(21-22)^.

Furthermore, one of the articles pointed out that health education provides health professionals with confidence to manage patients with covid-19^(29)^. Another included concepts for future projects, with increased virtual visibility of patients and the team through technological resources, helping to optimize the hospital unit^(27)^. A systematic review also listed detailed disinfection methods for surfaces, biological materials, air and PPE that are more efficient for eliminating the virus^(28)^. Finally, the last positive scenario showed preparation to manage the potential increase in hospitalizations, demonstrating that the facilities need to be adequate and equipped^(19)^.

In contrast, few studies had negative outcomes. One of them, carried out in the Emergency Department of a hospital, reported that contingency measures and change of plans were insufficient to manage hospital demand^(23)^. Another presented a more specific measure adopted in a cardiac surgery unit, which was nasal decolonization, which resulted in an increased risk of coughing and sneezing^(25)^. A study did not bring clear resolutions, results or outcomes^(15)^.

## DISCUSSION

The global health crisis caused by covid-19 has had negative impacts on various sectors, such as education, the economy and health. In the health systems of less developed countries, the repercussions were even more severe. For example, in Bangladesh, it was identified that the high demand for care made it difficult to access care in the public health system, making it necessary to transfer users to the private sector, which treated 77% of patients with SARS-CoV- 2^(30)^. Still in this sense, based on the results, a large number of studies carried out on the European continent can be observed, which is related to the fact that Europe was one of the epicenters of the virus at the beginning of the pandemic^(31)^.

According to the results of the present study, many health services have undergone changes in their care protocols in order to adapt to the new pandemic scenario to adequately promote patient safety. According to Rodriguez and Tantillo (2020)^(19)^, one of the major concerns of professionals is the organization of the flow of care, with regard to the follow-up of cases with suspected or confirmed covid-19.

In addition, it was identified that some institutions used specific environments that were isolated from other facilities to provide screening services. This is in line with the recommendation of the Ministry of Health (MS) that the flow of care should follow the priority of each patient, determined by the severity of the signs and symptoms presented^(32)^. In this sense, actions needed to be incorporated into health systems to promote patient safety in order to reduce adverse events. For this, there was the development of new policies and care protocols in the hospital sector^(33)^.

Faced with changes in care plans to achieve full patient safety during their care, more specific actions were needed, aimed at mitigating the adversities of the pandemic period in the hospital environment. Among the most frequently adopted security measures in the analyzed studies are contingency and reorganization of beds, rooms, and operating rooms; isolation and distancing practiced by patients and professionals; strict and adequate use of PPE; patient risk classification and postponement of elective surgeries; sorting reorganization; health education and training of professionals; swab in patients; disinfection of equipment and environments; and use of telehealth^(15 16,22 25,28)^.

Despite the adoption of all these measures and the mostly positive outcomes presented, a cross-sectional study^(34)^ in Croatia, carried out with doctors and nurses from a hospital on the front line of the pandemic, demonstrated that the areas with the lowest patient safety index were in the departments involved in the care of patients with covid-19. This suggested that a high workload was associated with a lower patient safety culture, with problems related to failures in team communication, shortage of professionals, as well as non-punitive response to errors and underreporting of events.

A qualitative study^(27)^ carried out in Australia found that, with effective communication and support to staff, it is possible to provide effective care to patients, despite the challenges posed by a pandemic. In addition, the creative use of technology to structure the workforce, disseminate information, keep staff up-to-date on guidelines, and promote engagement with patients’ families through videoconferencing was seen as facilitating care delivery^(35)^.

Regarding the health procedures affected by the pandemic period, it is undeniable that the challenges faced required an immediate creation of conditions to meet the various emerging needs of the population^(36)^. In the evaluated articles, it was observed that elective surgeries had to be postponed; and, when performed, prior isolation and swabs were required for all patients, in order to reduce the transmission of the virus and other opportunistic infections; in addition, outpatient consultations and screening began to be carried out through telemedicine and not in person^(25)^.

It is clear, therefore, that isolation is very effective in controlling the transmission of covid-19, although it can cause psychosocial deficits capable of lasting for a long period^(23,26)^. In hemodynamic facilities evaluated by one of the studies, it was found that, among the forms of containment of transmission, are continuous monitoring of isolation and early detection with rapid rRT-PCR test^(16)^. It is also noteworthy that isolation measures and establishment of exclusive areas became little used as studies demonstrated the main transmission routes and after the advent of vaccination^(16)^.

Accordingly, Landoas et al. (2021)^(24)^ argued in favor of monitoring nosocomial cases of covid-19 during the first wave, in order to determine the limit of protection of patients with the use of hygiene measures (such as hand asepsis, use of masks and measures contact blocking), which drastically reduced the transmission of the virus^(28)^.

From this perspective, before considering patients for elective surgery, it is necessary that they undergo a screening process. A limited number of surgeries are performed, giving preference to patients with a low risk of respiratory disease. Therefore, it is up to the multidisciplinary team to carefully assess which treatments can be postponed and which should be performed immediately, in addition to frequently reassessing the progression of the possible primary disease^(20,25)^.

In addition, part of the analyzed studies discussed technologies developed during the pandemic, in order to protect users and health professionals during procedures that required physical contact. For example, the study by Huddy et al. (2021)^(22)^ presents the results of a facility designed to provide emergency robotic surgery, in the safest possible way, in the early recovery period after the first peak of COVID-19 infection. Among the results of the study, the first ones suggest that one of the benefits of this installation may be shorter dwell times.

The application of technologies during the pandemic period was also part of care in the pediatric hospitalization area, including video technologies and measures to promote physical distancing and limit exposure. Furthermore, Penwill et al. (2021^(26)^ bring lessons that could be used to guide hospital leaders in possible future crises, such as the development of communication between the multidisciplinary team.

Still in this context, pediatric hospital resuscitation systems quickly adapted to the covid-19 pandemic, as shown by Morgan et al. (2020)^(18)^. Although institutions seek to create or adapt policies associated with team members, the use of PPE or airway management, in many situations the implemented changes needed to be modified in order to provide individualized care to each user.

### Study limitations

Despite the importance of the proposed theme, it was identified, as a limitation of the study, that the measures and protocols adopted are still not sufficient to promote patient safety in its entirety, especially in situations of lack of control, such as pandemics. In addition, it was found that the number of studies in the area is still scarce and that there is a need for work with more complex methods, such as randomized clinical trials, capable of showing in detail the actions to promote patient safety and their outcomes. Even so, it is possible that the methodological aspects have led to a limitation regarding the interpretation of the findings.

Furthermore, although the focus of the review is patient safety in the context of hospital units, the lack of strategies in other contexts, such as Primary Health Care, home care services, among others, can be identified as a limitation.

### Contributions to the Area

By exposing the main procedures adopted to promote patient safety, in addition to presenting the technology being developed in favor of health to mitigate the risk of contamination, this review brings together scientific evidence capable of assisting in the clinical management of patients hospitalized for covid-19. In this way, it seeks to contribute to the development of skills and abilities inherent to the work of nurses and other components of the multidisciplinary team; and serve as a basis for further research aimed at deepening some of the management highlighted.

Also, this study proposes proven effective ways to deal with infectious diseases in the hospital environment and even at home. Therefore, this work can be used as a subsidy for learning the techniques necessary in situations of outbreaks, epidemics or pandemics, in order to contribute directly to the improvement of quality of life.

## CONCLUSIONS

Reducing damage and commitment to the patient, the following actions mapped in this study proved to be very present and positive: contingency and reorganization of beds, rooms and operating rooms; isolation and distancing practiced by patients and professionals; use of PPE; patient risk classification; postponement of medical procedures; sorting reorganization; health education and training of professionals; performing swabs on patients; disinfection of equipment and environments; and use of telehealth.
